# Supplementary material for: Productivity, efficiency, and overall performance comparisons between attendings working solo versus attendings working with residents staffing models in an emergency department: A Large-Scale Retrospective Observational Study
Source: PLoS One. 2020 Feb 5;15(2):e0228719. doi: 10.1371/journal.pone.0228719 (PMC7001986; doi:10.1371/journal.pone.0228719)
Supplement: S1 Appendix — (DOCX) [file pone.0228719.s001.docx]

S1 Appendix

- 1. Detail Explanation of Emergency Severity Index (ESI)

In this study, an ESI version 4 was used. ESI includes a five-level acuity scale applied upon each patient’s completed triage at the Emergency Department (ED). These five-levels of acuity range from ESI 1 (most urgent) to ESI-5 (least urgent). A detail explanation of ESI can be found at The Agency of Healthcare Research and Quality (AHRQ) website via the following links:

<https://www.ahrq.gov/professionals/systems/hospital/esi/index.html>

<https://www.ahrq.gov/sites/default/files/wysiwyg/professionals/systems/hospital/esi/esialgorithm_card.pdf>


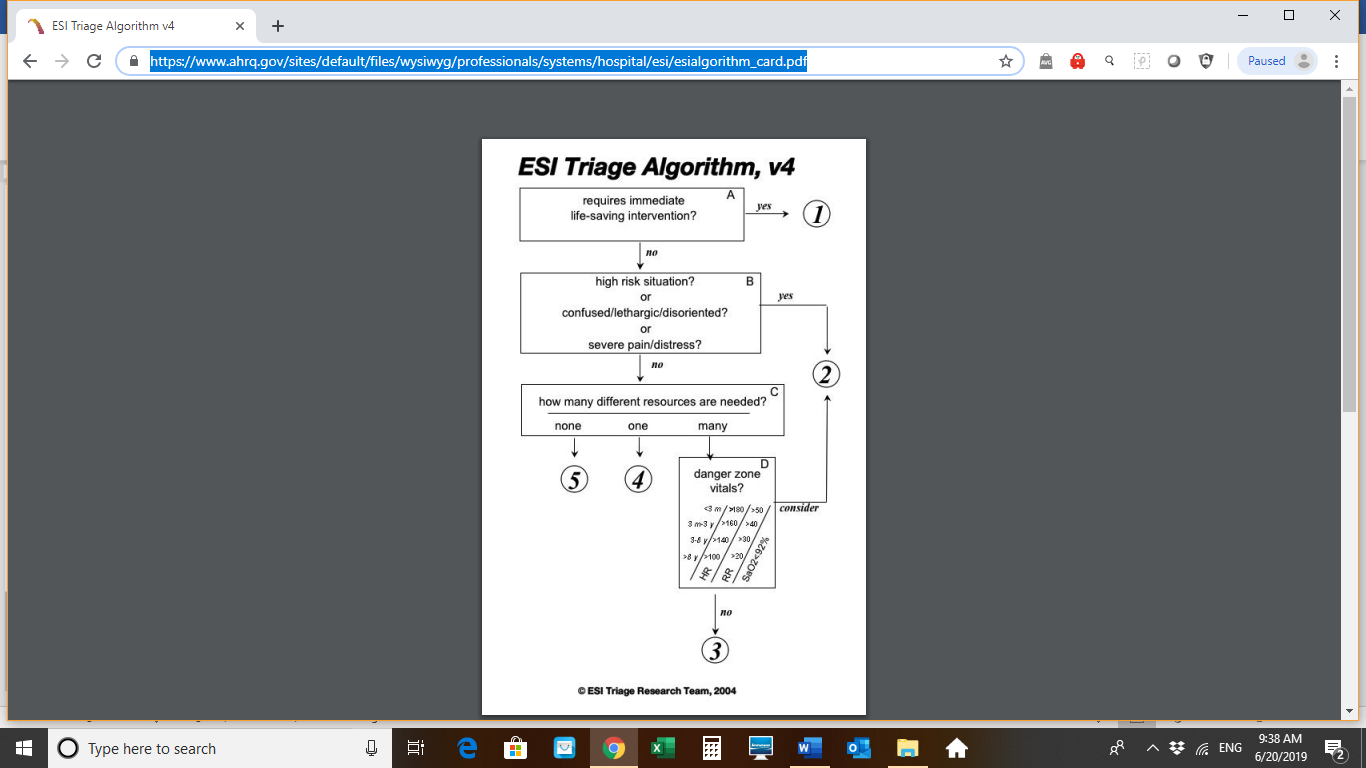


- 1. Detail Variables Explanation

ED Length of Stay (LOS) is defined as the time in minutes beginning at the point the patient is initially arrived and registered into the EMR at the ED and ending at the point that the patient physically leaves the ED indicating closure of that specific encounter.

Provider-to-Disposition time (PDT) is defined as the time interval documented in the EMR beginning at the point when the patient is initially seen and evaluated by a provider and ending at the point when the disposition decision is made.

The number of new patients per hour is defined as the number of new patients assigned to individual providers within a one-hour block (e.g., 0200 to 0259).

ED crowding is measured using the NEDOCS score (National Emergency Department Over-Crowding Study) upon each patient’s arrival to the ED (see below). The definitions of these variables are consistent with those previously published (Welch SJ, Asplin BR, Stone-Griffith S, Davidson SJ, Augustine J, Schuur J: **Emergency department operational metrics, measures and definitions: results of the Second Performance Measures and Benchmarking Summit.** *Ann Emerg Med* 2011, **58:** 33-40.). NEDOCS score equals or less than 100 (NEDOCS≤100) is considered ED not crowding, NEDOCS score less than 140 but greater than 100 (100<NEDOCS<140) is considered ED crowding, and NEDOCS score equal or greater than 140 (NEDOCS≥140) is considered ED overcrowding.

Detail Explanation of NEDOCS Score Calculation

| Variables | Definition |
| --- | --- |
| NEDOCS = 85.8T + 600B + 5.64W + 0.93L + 13.4C -20 | |
| T | The total number of ED patients collected divided by the number of licensed beds at the time a score is calculated |
| B | The number of admitted patients/number of hospital beds at the time a score is calculated |
| W | Longest wait time in hours for patients in the waiting room at the time a score is calculated |
| L | Longest time in hours since registration among boarding patients at the time a score is calculated |
| C | Number of critical care patients at the time a score is calculated. Typically, this is a site-specific variable which usually refers to patients that require one-to-one nursing care. In the study ED, critical care patients are defined as ICU patients and ICU consulted patients including but not limited to patients on mechanical ventilators, receiving tPA, diagnosed with septic shock, critical trauma patients, and patients requiring conscious sedation at the time a score is calculated, etc. |

1.3 Detail Explanation of Attending Performance Index (API)

In our study, API is calculated for each patient encounter. That is, a unique API is associated with each patient treated by an Attending to demonstrate the Attending’s performance per individual patient encounter. Each API is calculated based on three metrics: 1) the number of new patients seen per hour by the Attending; 2) PDT of a patient encounter, and 3) patient ESI. The number of new patients per hour is a productivity metric that measures the number of new patients treated by the Attending during an hour (the larger the number of patients per hour, the greater the productivity). PDT is the efficiency metric of the Attending for a particular patient (the shorter the PDT, the higher the efficiency). Individual patient ESI is used for adjusting patient heterogeneity. For example, if two patients treated by the same Attending had the same PDT and number of patients seen per hour, and the only difference is that the first patient is triaged as an ESI Level 1, and the second patient is triaged as an ESI Level 5, then Attending performance, measured by API, for treating the first patient (ESI = 1) is better (higher score) than that for treating the second patient (ESI = 5). We use three tiers of API to assess overall Attending performance, first quartile (Q1), median, and third quartile (Q3), which account for 25%, 50%, and 75% of the patients treated by the Attending, respectively.

*Attending Performance Index* =

*Number of New Patients per Hour per Attending*

*(Acuity Level)^2^ X Provider-to-Disposition Time (hours)*
