# Supplementary material for: Productivity, efficiency, and overall performance comparisons between attendings working solo versus attendings working with residents staffing models in an emergency department: A Large-Scale Retrospective Observational Study
Source: PLoS One. 2020 Feb 5;15(2):e0228719. doi: 10.1371/journal.pone.0228719 (PMC7001986; doi:10.1371/journal.pone.0228719)
Supplement: S2 Appendix — (DOCX) [file pone.0228719.s002.docx]

S2 Appendix Daily Patient Volume as Measured During

Time Interval 2300 (Previous Day) through 1500 (Next Day)

| Time Intervals | Total Number of Patients Presenting at ED |
| --- | --- |
| Sun 2300 through Mon 1500 | 29,535 |
| Mon 2300 through Tue 1500 | 33,705 |
| Tue 2300 through Wed 1500 | 33,384 |
| Wed 2300 through Thu 1500 | 32,989 (Attending solo) |
| Thu 2300 through Fri 1500 | 31,831 |
| Fri 2300 through Sat 1500 | 31,426 |
| Sat 2300 through Sun 1500 | 30,533 |
