# Supplementary material for: Productivity, efficiency, and overall performance comparisons between attendings working solo versus attendings working with residents staffing models in an emergency department: A Large-Scale Retrospective Observational Study
Source: PLoS One. 2020 Feb 5;15(2):e0228719. doi: 10.1371/journal.pone.0228719 (PMC7001986; doi:10.1371/journal.pone.0228719)
Supplement: S3 Appendix — (DOCX) [file pone.0228719.s003.docx]

S3 Appendix – Productivity Comparisons Between Attendings

Working Solo versus Attendings Working with Residents

|  | Original Data | | | Propensity Score Matching Data | | |
| --- | --- | --- | --- | --- | --- | --- |
|  | Attendings  Solo  Median n (IQR) | Attendings with Residents  Median n (IQR) |  | Attendings  Solo  Median n (IQR) | Attendings with Residents  Median n (IQR) |  |
| Attending-1 | 2 (1, 3) | 3 (2, 4) |  | 2 (1, 3) | 3 (2,4) |  |
| Attending-2 | 2 (1, 2) | 3 (2, 4) |  | 2 (1, 2) | 3 (2, 4) |  |
| Attending-3 | 2 (1, 3) | 3 (2, 4) |  | 2 (1, 3) | 3 (2, 4) |  |
| Attending-4 | 2 (1, 4) | 3 (2, 4) |  | 2 (1, 4) | 3 (2, 4) |  |
| Attending-5 | 3 (2, 4) | 3 (2, 4) |  | 3 (2, 4) | 3 (2, 4) |  |
| Attending-6 | 2 (2, 4) | 3 (2, 4) |  | 2 (2, 4) | 3 (2, 4) |  |
| Attending-7 | 3 (2, 4) | 3 (2, 5) |  | 3 (2, 4) | 3 (2, 5) |  |
| Attending-8 | 2 (2, 3) | 3 (2, 4) |  | 2 (2, 3) | 3 (2, 4) |  |
| Attending-9 | 3 (2, 4) | 3 (2, 4) |  | 3 (2, 4) | 3 (2, 4) |  |
| Attending-10 | 3 (2, 4) | 3 (2, 4) |  | 3 (2, 4) | 3 (2, 4) |  |
| Attending-11 | 3 (2, 5) | 3 (2, 5) |  | 3 (2, 5) | 3 (2, 4) |  |
| Attending-12 | 3 (2, 4) | 3 (2, 5) |  | 3 (2, 4) | 3 (2, 5) |  |
| Attending-13 | 3 (2, 4) | 3 (2, 4) |  | 3 (2, 4) | 3 (2, 4) |  |
| Attending-14 | 3 (2, 4) | 3 (2, 5) |  | 3 (2, 4) | 3 (2, 4) |  |
| Attending-15 | 3 (2, 4) | 3 (2, 4) |  | 3 (2, 4) | 3 (2, 4) |  |

Abbreviations: IQR, Interquartile Range; n, number of new patients seen per Attending physician per hour used for Productivity analysis.
