# Supplementary material for: Productivity, efficiency, and overall performance comparisons between attendings working solo versus attendings working with residents staffing models in an emergency department: A Large-Scale Retrospective Observational Study
Source: PLoS One. 2020 Feb 5;15(2):e0228719. doi: 10.1371/journal.pone.0228719 (PMC7001986; doi:10.1371/journal.pone.0228719)
Supplement: S4 Appendix — (DOCX) [file pone.0228719.s004.docx]

S4 Appendix Efficiency (Provider to Disposition Time) Comparisons Between

Attendings Working Solo versus Attendings Working with Residents

|  | Original Data | | | Propensity Score Matching Data | | |
| --- | --- | --- | --- | --- | --- | --- |
|  | Attendings  Solo  Median PDT (IQR) | Attendings with Residents  Median PDT (IQR) |  | Attendings  Solo  Median PDT (IQR) | Attendings with Residents  Median PDT (IQR) |  |
| Attending-1 | 3.2 (2.2, 4.3) | 3.2 (2.1, 4.6) |  | 3.2 (2.2, 4.3) | 3.5 (2.3, 4.9) |  |
| Attending-2 | 2.8 (1.9, 3.9) | 2.9 (1.8, 4.1) |  | 2.8 (1.9, 3.9) | 3.2 (2.1, 4.5) |  |
| Attending-3 | 2.9 (2.0, 3.8) | 2.9 (1.8, 4.1) |  | 2.9 (2.0, 3.8) | 3.0 (2.1, 4.4) |  |
| Attending-4 | 2.9 (1.8, 4.3) | 2.9 (1.9, 4.2) |  | 2.9 (1.8, 4.3) | 3.0 (2.0, 4.3) |  |
| Attending-5 | 3.2 (2.1, 4.6) | 2.8 (1.8, 4.1) |  | 3.2 (2.1, 4.6) | 2.8 (2.0, 4.0) |  |
| Attending-6 | 2.7 (1.6, 4.1) | 2.8 (1.7, 4.0) |  | 2.7 (1.6, 4.1) | 3.0 (1.8, 4.4) |  |
| Attending-7 | 2.9 (1.8, 4.2) | 2.6 (1.6, 3.7) |  | 2.9 (1.8, 4.2) | 3.1 (1.9, 4.4) |  |
| Attending-8 | 2.4 (1.5, 3.3) | 2.6 (1.6, 3.8) |  | 2.4 (1.5, 3.3) | 2.8 (1.8, 3.9) |  |
| Attending-9 | 2.1 (1.3, 2.9) | 2.5 (1.6, 3.7) |  | 2.1 (1.3, 2.9) | 2.6 (1.8, 3.8) |  |
| Attending-10 | 2.0 (1.2, 3.3) | 2.9 (1.8, 4.2) |  | 2.0 (1.2, 3.3) | 3.2 (2.3, 4.3) |  |
| Attending-11 | 1.8 (1.0, 3.1) | 2.6 (1.6, 3.7) |  | 1.8 (1.0, 3.1) | 2.6 (1.7, 4.0) |  |
| Attending-12 | 2.2 (1.5, 3.3) | 2.7 (1.7, 3.8) |  | 2.2 (1.5, 3.3) | 3.2 (2.0, 4.5) |  |
| Attending-13 | 2.1 (0.7, 3.5) | 2.7 (1.7, 4.0) |  | 2.1 (0.7, 3.5) | 2.8 (1.7, 4.2) |  |
| Attending-14 | 1.8 (1.1, 2.7) | 2.5 (1.6, 3.6) |  | 1.8 (1.1, 2.7) | 2.8 (1.7, 3.9) |  |
| Attending-15 | 1.8 (1.1, 2.9) | 2.8 (1.9, 4.0) |  | 1.8 (1.1, 2.9) | 2.9 (2.1, 4.0) |  |

Abbreviations and definitions: IQR, Interquartile Range; PDT, Provider-to-Disposition; Provider-to-Disposition time in hours used as efficiency analysis.
