# Supplementary material for: Productivity, efficiency, and overall performance comparisons between attendings working solo versus attendings working with residents staffing models in an emergency department: A Large-Scale Retrospective Observational Study
Source: PLoS One. 2020 Feb 5;15(2):e0228719. doi: 10.1371/journal.pone.0228719 (PMC7001986; doi:10.1371/journal.pone.0228719)
Supplement: S5 Appendix — (DOCX) [file pone.0228719.s005.docx]

S5 Appendix Overall Performance Comparisons Between

Attendings Working Solo versus Attendings Working with Residents

|  | Original Data | | | Propensity Score Matching Data | | |
| --- | --- | --- | --- | --- | --- | --- |
|  | Attendings  Solo  Median API (IQR) | Attendings with Residents  Median API (IQR) | Differences | Attendings  Solo  Median API (IQR) | Attendings with Residents  Median API (IQR) | Differences |
| Attending-1 | 0.10 (0.05, 0.18) | 0.12 (0.06, 0.23) | + | 0.10 (0.05, 0.18) | 0.11 (0.06, 0.21) | + |
| Attending-2 | 0.10 (0.05, 0.18) | 0.14 (0.07, 0.26) | + | 0.10 (0.05, 0.18) | 0.11 (0.07, 0.20) | + |
| Attending-3 | 0.11 (0.06, 0.20) | 0.16 (0.08, 0.31) | + | 0.11 (0.06, 0.20) | 0.15 (0.07, 0.25) | + |
| Attending-4 | 0.12 (0.06, 0.27) | 0.15 (0.08, 0.29) | + | 0.12 (0.06, 0.27) | 0.13 (0.07, 0.24) | + |
| Attending-5 | 0.12 (0.07, 0.25) | 0.16 (0.09, 0.32) | + | 0.12 (0.07, 0.25) | 0.14 (0.08, 0.27) | + |
| Attending-6 | 0.13 (0.07, 0.29) | 0.16 (0.09, 0.31) | + | 0.13 (0.07, 0.29) | 0.15 (0.08, 0.26) | + |
| Attending-7 | 0.14 (0.07, 0.27) | 0.20 (0.10, 0.41) | + | 0.14 (0.07, 0.27) | 0.15 (0.08, 0.30) | + |
| Attending-8 | 0.16 (0.09, 0.35) | 0.18 (0.09, 0.36) | + | 0.16 (0.09, 0.35) | 0.16 (0.08, 0.28) | +/- |
| Attending-9 | 0.18 (0.10, 0.38) | 0.18 (0.10, 0.36) | +/- | 0.18 (0.10, 0.38) | 0.16 (0.08, 0.31) | - |
| Attending-10 | 0.22 (0.12, 0.50) | 0.16 (0.08, 0.32) | - | 0.22 (0.12, 0.50) | 0.15 (0.08, 0.28) | - |
| Attending-11 | 0.23 (0.11, 0.67) | 0.20 (0.10, 0.42) | - | 0.23 (0.11, 0.67) | 0.18 (0.09, 0.33) | - |
| Attending-12 | 0.23 (0.11, 0.47) | 0.19 (0.10, 0.39) | - | 0.23 (0.11, 0.47) | 0.17 (0.09, 0.31) | - |
| Attending-13 | 0.23 (0.11, 0.64) | 0.17 (0.09, 0.34) | - | 0.23 (0.11, 0.64) | 0.14 (0.08, 0.29) | - |
| Attending-14 | 0.24 (0.13, 0.54) | 0.21 (0.11, 0.42) | - | 0.24 (0.13, 0.54) | 0.15 (0.08, 0.28) | - |
| Attending-15 | 0.24 (0.13, 0.38) | 0.16 (0.09, 0.29) | - | 0.24 (0.13, 0.38) | 0.14 (0.08, 0.26) | - |

Abbreviations and definitions: IQR, Interquartile Range; Performance measure = Attending Performance Index [(number of new patients per hour seen by a provider)/((patient acuity level determined by ESI)^2^ x (provider to disposition time in hours))]. +: increased overall performance when individual attending works with residents compared with same attending working solo; -: decreased overall performance when individual attending works with residents compared with same attending working solo; +/- : no overall performance differences for a given attending between the two groups.
