# Supplementary figures and images for: Productivity, efficiency, and overall performance comparisons between attendings working solo versus attendings working with residents staffing models in an emergency department: A Large-Scale Retrospective Observational Study
Source: PLoS One. 2020 Feb 5;15(2):e0228719. doi: 10.1371/journal.pone.0228719 (PMC7001986; doi:10.1371/journal.pone.0228719)

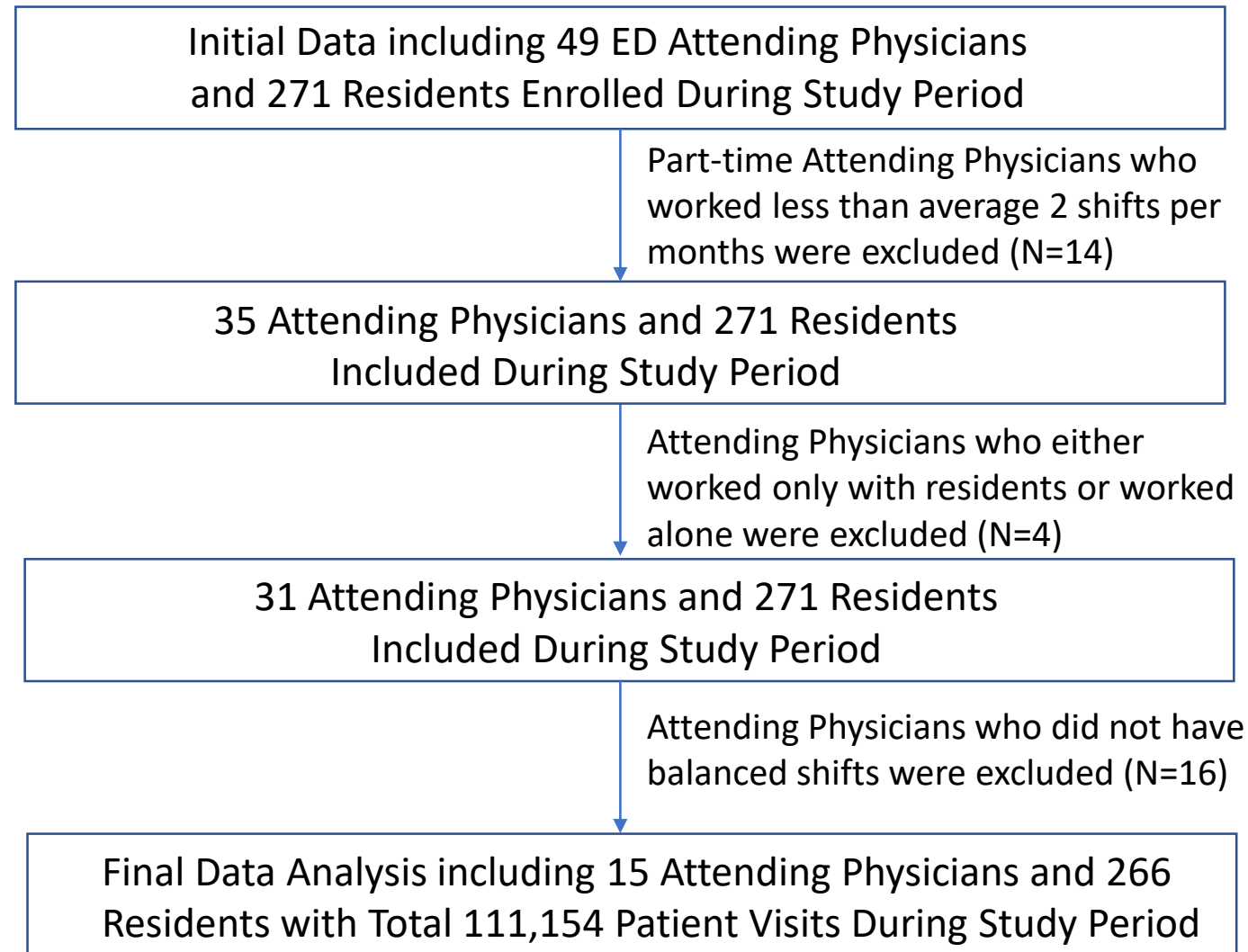

S1 Appendix Fig – Study Flow Diagram

Supplement: S1 Appendix Fig — (PDF) [file pone.0228719.s006.pdf]
