# Supplementary material for: Productivity, efficiency, and overall performance comparisons between attendings working solo versus attendings working with residents staffing models in an emergency department: A Large-Scale Retrospective Observational Study
Source: PLoS One. 2020 Feb 5;15(2):e0228719. doi: 10.1371/journal.pone.0228719 (PMC7001986; doi:10.1371/journal.pone.0228719)
Supplement: Attachment — Submitted filename: response to editor and reviewers comments (R1).docx [file pone.0228719.s007.docx]

**AUTHORS RESPONSE TO EDITORS AND REVIEWERS**

As requested, we have included the original letter and comments with our point by point response in red colored font.

PONE-D-19-26595
Productivity, Efficiency, and Overall Performance Comparisons Between Solo Attending Versus Attending with Residents Staffing Models in an Emergency Department
PLOS ONE

Dear Dr. Wang,

Thank you for submitting your manuscript to PLOS ONE. After careful consideration, we feel that it has merit but does not fully meet PLOS ONE’s publication criteria as it currently stands. Therefore, we invite you to submit a revised version of the manuscript that addresses the points raised during the review process.

We would appreciate receiving your revised manuscript by Dec 27 2019 11:59PM. When you are ready to submit your revision, log on to https://www.editorialmanager.com/pone/ and select the 'Submissions Needing Revision' folder to locate your manuscript file.

If you would like to make changes to your financial disclosure, please include your updated statement in your cover letter.

To enhance the reproducibility of your results, we recommend that if applicable you deposit your laboratory protocols in protocols.io, where a protocol can be assigned its own identifier (DOI) such that it can be cited independently in the future. For instructions see: <http://journals.plos.org/plosone/s/submission-guidelines#loc-laboratory-protocols>

Please include the following items when submitting your revised manuscript:

- A rebuttal letter that responds to each point raised by the academic editor and reviewer(s). This letter should be uploaded as separate file and labeled 'Response to Reviewers'.
- A marked-up copy of your manuscript that highlights changes made to the original version. This file should be uploaded as separate file and labeled 'Revised Manuscript with Track Changes'.
- An unmarked version of your revised paper without tracked changes. This file should be uploaded as separate file and labeled 'Manuscript'.

Please note while forming your response, if your article is accepted, you may have the opportunity to make the peer review history publicly available. The record will include editor decision letters (with reviews) and your responses to reviewer comments. If eligible, we will contact you to opt in or out.

We look forward to receiving your revised manuscript.

Kind regards,

Andrew Carl Miller
Academic Editor
PLOS ONE

Journal Requirements:

When submitting your revision, we need you to address these additional requirements.

Please ensure that your manuscript meets PLOS ONE's style requirements, including those for file naming. The PLOS ONE style templates can be found at

http://www.journals.plos.org/plosone/s/file?id=wjVg/PLOSOne_formatting_sample_main_body.pdf and http://www.journals.plos.org/plosone/s/file?id=ba62/PLOSOne_formatting_sample_title_authors_affiliations.pdf

1. We note that you have indicated that data from this study are available upon request. PLOS only allows data to be available upon request if there are legal or ethical restrictions on sharing data publicly. For information on unacceptable data access restrictions, please see http://journals.plos.org/plosone/s/data-availability#loc-unacceptable-data-access-restrictions.

In your revised cover letter, please address the following prompts:

a) If there are ethical or legal restrictions on sharing a de-identified data set, please explain them in detail (e.g., data contain potentially identifying or sensitive patient information) and who has imposed them (e.g., an ethics committee). Please also provide contact information for a data access committee, ethics committee, or other institutional body to which data requests may be sent.

Response: Yes, our data contain identifiable patient information including patient name, age, and admission date/time. This project was approved by the local Institutional Review Board (IRB). We provided information of the contact person to whom data requests may be sent.

b) If there are no restrictions, please upload the minimal anonymized data set necessary to replicate your study findings as either Supporting Information files or to a stable, public repository and provide us with the relevant URLs, DOIs, or accession numbers. Please see http://www.bmj.com/content/340/bmj.c181.long for guidelines on how to de-identify and prepare clinical data for publication. For a list of acceptable repositories, please see http://journals.plos.org/plosone/s/data-availability#loc-recommended-repositories.

We will update your Data Availability statement on your behalf to reflect the information you provide.

2. Please include captions for your Supporting Information files at the end of your manuscript, and update any in-text citations to match accordingly. Please see our Supporting Information guidelines for more information: <http://journals.plos.org/plosone/s/supporting-information>.

Response: Yes, we revised and included captions for our supplemental figure at the end of our manuscript.


[Note: HTML markup is below. Please do not edit.]

Reviewers' comments:

Reviewer's Responses to Questions

**Comments to the Author**

1. Is the manuscript technically sound, and do the data support the conclusions?

The manuscript must describe a technically sound piece of scientific research with data that supports the conclusions. Experiments must have been conducted rigorously, with appropriate controls, replication, and sample sizes. The conclusions must be drawn appropriately based on the data presented.

Reviewer #1: No

Reviewer #2: Yes

2. Has the statistical analysis been performed appropriately and rigorously?

Reviewer #1: I Don't Know

Reviewer #2: Yes

3. Have the authors made all data underlying the findings in their manuscript fully available?

The [PLOS Data policy](http://www.plosone.org/static/policies.action#sharing) requires authors to make all data underlying the findings described in their manuscript fully available without restriction, with rare exception (please refer to the Data Availability Statement in the manuscript PDF file). The data should be provided as part of the manuscript or its supporting information, or deposited to a public repository. For example, in addition to summary statistics, the data points behind means, medians and variance measures should be available. If there are restrictions on publicly sharing data—e.g. participant privacy or use of data from a third party—those must be specified.

Reviewer #1: Yes

Reviewer #2: Yes

4. Is the manuscript presented in an intelligible fashion and written in standard English?

PLOS ONE does not copyedit accepted manuscripts, so the language in submitted articles must be clear, correct, and unambiguous. Any typographical or grammatical errors should be corrected at revision, so please note any specific errors here.

Reviewer #1: No

Reviewer #2: Yes

5. Review Comments to the Author

Please use the space provided to explain your answers to the questions above. You may also include additional comments for the author, including concerns about dual publication, research ethics, or publication ethics. (Please upload your review as an attachment if it exceeds 20,000 characters)

Reviewer #1:

1. -Based on the data presented in the results section of the abstract, the conclusion that “increased productivity […] often occurs among attendings working with residents” isn’t supported by the data cited as you cited identical median numbers. If other data shows this, cite that instead. Also I would take out the word “often.”

Response: Although no obvious MEDIAN differences were observed between Attendings working solo versus working with Residents by reviewing the median (IQR) of the performance measures, the Wilcoxon-Rank Sum test did show statistically significant differences in their means (*p-value* <0.001). Wilcoxon-Rank Sum test is a non-parametric statistical test for comparing means of two samples that may not be normally distributed. Sorry for the confusion. In order to better interpret our data, we revised and added the mean (SD) along with the median (IQR) in Table 2 (statistically significant differences between these two groups were found). We used mean (SD) of productivity and efficiency reported in the abstract (Results section). In addition, we deleted “often” under the Conclusions section of the abstract.

2. First paragraph: However, both are affected by multiple factors including ED crowding, patient acuity, (AND) supervision of Residents and Medical Students. [5-7].
   Second paragraph: “Whereas, another study determined that ED LOS was not significantly affected by the presence or total number of trainees in the ED [11]” is a sentence fragment. Change to “However, another study determined that ED LOS was not significantly affected by the presence or total number of trainees in the ED [11].”

Response: Yes, we revised and added “and” between “patient acuity,” and “supervision of Residents and Medical Students.”. We also revised, and changed to “However, another study determined that ED LOS was not significantly affected by the presence of total number of trainees in the ED [11].” (see introduction section, the first and second paragraphs).

3. Second paragraph: “At present, few studies compare provider productivity and efficiency between Attendings working solo versus Attendings working with Residents.” How does this study differ from or add to what we already have in the literature on this?

Response: Thanks for reviewer’s valued comment. We revised and added the importance of this study to address how it differs from other studies reported in the literature (see below). It was inserted in the second paragraph of the Introduction section.

“At present, only a few studies compare provider productivity and efficiency between Attendings working solo versus Attendings working with Residents, and none of them examine the differences in productivity and efficiency of individual Attendings within these two groups.”

4. Third paragraph: “Overall provider performance may be gained (change to “calculated” or “defined”)by combining productivity and efficiency arriving at a composite measure [13].” This paragraph at the end of the introduction does not make sense all by itself. If you want to introduce a new metric, I would do it in the first paragraph where you start defining the metrics used.

Response: Yes, we revised and changed “gained” to “calculated”. We realize this was confusing. Sorry about that. The whole paragraph mentioned above is indeed part of the literature review. We intend to report a composite index was developed previously to measure providers’ overall performance; however, this index lacks external validation. In terms of the flow of the paper, we think it would be appropriate to describe it here together with other existing work.

”Productivity and efficiency are often consistent when used to evaluate provider operational performance (i.e., efficient providers are also productive or vice versa). However, it is not uncommon to observe some inconsistencies (e.g., high productivity but low efficiency), thus creating a significant challenge to understanding provider overall operational performance. To overcome this challenge, prior research introduced a composite index, which was calculated by combining productivity and efficiency [13]. However, a major shortcoming of said calculation is the lack of external validation.”

5. The paper switches frequently between passive and active tense; this needs to be more consistent.

Response: Yes, we revised the entire manuscript to be more consistent.

6. In the results section, the conclusion that “Analysis of productivity reveals more patients per hour were seen by Attendings working with Residents than Attendings working solo (Table 2).” Is not supported by the data cited which shows identical median patient numbers for both groups.

Response: Again, sorry for the confusion. We revised it and added both median (IQR) and mean (SD) of productivity and efficiency of attendings solo versus attendings working with residents (see revised Table 2).

7. In the results section, why was the cut off of 0.18 used for the API for high index or low index categorization? Has this been looked at and defined in the past?

Response: No, the cutoff of 0.18 has not been reported in the past. This number was derived by interpreting this study’s data at the individual provider level (see Append Table 5). We revised and added explanations in the Methods and Results sections as follows:

1. In the Methods section (last paragraph of the Methods section: data analysis)

“To assess overall performance changes, the APIs of individual Attendings were calculated and compared between individual Attendings working solo and working with Residents. Scant literature exists regarding overall performance measurement of Attendings and no quantitative benchmark value has been established to date indicating whether calculated API is predictive of Attending performance. Therefore, a threshold value API was determined in this study based on the changes of individual Attending APIs working with or without Residents. Indeed, this threshold value was a cutoff value at which different patterns of Attending overall performance were observed when working solo and working with Residents.”

2. In the Results section (third paragraph of the Results section)

“Additionally, when Attending productivity, efficiency, and overall performance are measured at an individual Attending level, the case of increased productivity with decreased efficiency is observed for most individual Attendings when working with Residents compared to working solo (Appendix-3 and Appendix-4). All 15 Attendings are divided into two groups in terms of their solo performance. Higher Attending performance indexes (APIs) are observed among individual Attendings when working with Residents if their solo API < 0.18. On the contrary, lower APIs were noted among individual Attendings whose solo API ≥ 0.18 when working with Residents (Appendix-5). This pattern remained when confounding factors were adjusted using the 1:1 propensity score matching model (Table 3). Therefore, a threshold API (high API ≥ 0.18 and low API < 0.18) of individual Attending performance was determined in this study.”

8. Discussion: First sentence “Our study found increased productivity with decreased efficiency among Attendings working with Residents.” Is not supported by the data presented which cites identical median productivity numbers. How are you getting to this conclusion?

Response: As we addressed before, we added our mean (SD) of productivity and efficiency to both attendings working solo and attendings working with resident groups. Both showed statistically significant differences. (see response of comment #1 and Table 2)

9. “Though the majority of high acuity patients (ESI 1-2-3) were seen by Attendings regardless of whether they worked solo or with Residents, differences occurred between these two groups.” What differences are these?

Response: Sorry for the confusion. We revised this sentence and added “productivity and efficiency differences”. Please see the revised following:

“Though the majority of high acuity patients (ESI 1-2-3) were seen by Attendings regardless of whether they worked solo or with Residents, productivity and efficiency differences occurred between these two groups.”

10. Discussion:
    “Therefore, to simplify this study, the number of new patients per hour as opposed to RVUs per hour was used as our Attending productivity measurement [4,15]. On the other hand, we used PDT instead of patient LOS for provider efficiency measurements because LOS is often affected by different system and patient associated variables serving as meaningful confounders (e.g., ED crowding, waiting room time, etc.) [16,17]. Unlike LOS, PDT is more directly affected by a given provider during the decision-making process and resultant downstream diagnostic and therapeutic resource needs [4,13]. Therefore, PDT delivers better interpretive quality regarding Attending efficiency.” All of this likely would do better in the methods section as it does not discuss the results of your study.

Response: Thanks for reviewer’s valued comment. We considered it necessary to discuss the reason we use the number of new patients per hour as a parameter for provider productivity measurement in this study instead of using others (e.g. RVU, number of new patients per shift). Same as efficiency measurement, we addressed why we use PDT instead of patient LOS as the parameter for efficiency measurement. We believe a discussion on the variety of operational metrics used for productivity/efficiency measurements might be suitably placed in the Discussion section. However, we understand the confusion, we revised our discussion as follows (see the second paragraph under Discussion section).

“Productivity and efficiency can be affected by differences in patient acuity. The study ED preferentially flows patients such that Attendings working solo and Attendings working with Residents provide high acuity care and the APP staff provide low acuity care. Though the majority of high acuity patients (ESI 1-2-3) were seen by Attendings regardless of whether they worked solo or with Residents, productivity and efficiency differences occurred between these two groups. Therefore, it is necessary to use propensity matching to minimize potential confounder effects in this study. When propensity score matching was applied, the median number of new patients per hour seen by either Attendings working solo or Attendings working with Residents reveals no changes both at the group (Table 2) and individual (Table 3) levels, indicating that patient acuity level has no effect on provider productivity measurement. In addition, different metrics have been used to measure provider productivity in the literature, including RVUs or number of new patients per shift. However, RVUs can be significantly affected based on encounter documentation alone. Our providers usually spend extra time outside of their clinical shift to complete documentation, thus it is extremely difficult and inaccurate to add extra time for documentation when deriving the provider productivity calculation. Moreover, providers working at the study ED typically work different length shifts (e.g., 8h, 9h, 10h, or 12h shifts), serving as a potential confounder that fails to compare provider productivity on a common basis when number of new patients per *shift* is used. Therefore, to simplify this study, the number of new patients per *hour*, as opposed to RVUs per hour and/or patients per *shift*, was used as our Attending productivity measurement [4,15]. On the other hand, we used PDT instead of patient LOS for provider efficiency measurement because LOS is often affected by different system- and patient-associated variables, which could be potential confounders in the context of provider efficiency (e.g., ED crowding, waiting room time, etc.) [16,17]. Unlike LOS, PDT is more directly affected by a given provider during the decision-making process impacting downstream diagnostic and therapeutic resource needs turnaround intervals [4,13]. Therefore, PDT delivers better interpretive quality regarding Attending efficiency. Ideally, a higher number of new patients per hour (high productivity) along with a lower PDT per patient (high efficiency) is considered the most desirable overall provider performance outcome combination. A balanced provider performance index was therefore used in this study [13]. ”

Reviewer #2: General:
11. - Inserting line numbers will make it easier for reviewers to provide more focused feedback.

Response: yes, we inserted line numbers to the manuscript.

12. - Please be sure to list the appropriate guideline used and provide citation. For example:
von Elm E, Altman DG, Egger M, Pocock SJ, Gotzsche PC, Vandenbroucke JP. The Strengthening the Reporting of Observational Studies in Epidemiology (STROBE) Statement: guidelines for reporting observational studies. PLoS Med. 2007;4(10):e296. PMID: 17941714
Response: Yes, we revised and added the appropriate guideline under the method section. (see method section, under reporting guideline)

13. Title:
- Indicate the study’s design with a commonly used term in the title or the abstract
Response: Yes, we revised the title as the following:

“Productivity, Efficiency, and Overall Performance Comparisons Between Solo Attending Versus Attending with Residents Staffing Models in an Emergency Department: A Large-scale Retrospective Observational Study”

14. Abstract:
- Please reposition the headings Methods, Results, and Conclusions so that they are not contiguous with the preceding paragraph.

Response: Yes, they are revised.

15. Introduction:
- Pg 4: Resident efficiency has been reported as less than that of APPs. How does this compare with the number of bounce-back or repeat visits? The number of M&M cases? APP practice environments vary greatly; are the APPs in this comparison practicing autonomously (attending consult available but not required), or do they have to present their cases to an attending still? Were they APPs who had done an EM “residency” or practicing straight out of school?

Response: Thanks for reviewer’s valued comment. Patients who had bounce-back or repeated visits will be counted as another patient encounter and treated as a new patient. In this study, we did not specifically address the number of M&M cases. We understand APP practice differs in different environments. Therefore, in this study, we excluded patients seen by APPs. In the study ED, APPs see patients independently and can disposition patients by themselves without the need to notify attending physicians. Under very rare circumstances, an APP might call an attending physician to get a second opinion on their patients since APPs only see low acuity patients. All APPs will need ED experience before they work at the study ED. We realize such uncertainty, we revised and addressed these under the Methods section.

16. - Same line as above: is there any evidence that the APPs document better, thereby billing at a higher level and generating more RVUs? To aid discussion of these points, one my consider incorporating the reference by McDonnell into the discussion (PMID: 25654675 DOI: 10.1097/PEC.0000000000000349).
Response: Since this project mainly discussed attending and resident productivity, efficiency and their performance, we did not include APPs in this study.

17. Methods:
- The ESI is a poor judge of illness severity. From the EMR one may be able to obtain the necessary info to determine illness severity using a validated tool such as Charlson Comorbidity Index, or 3M-APR-DRG. This would be helpful for inter-group comparison.

Response: Thanks for reviewer’s valued comment. We understand that ESI is a poor indicator for illness severity. Unfortunately, due to the nature of this study’s design, we are unable to add CCI or APR-DRG to the final analysis. We revised and addressed these in our limitation section. (see the fifth paragraph under discussion section)

18. - Explain how the study size was arrived at. Provide a calculation to justify sample size & method used.

Response: This is a retrospective study and our intent is to include all patients seen by attendings and residents during the study period. Therefore, we did not perform the sample size estimation in this study.

19. - STATA 14.2 software. It is convention to list the manufacturer & location in parentheses after the name. Additional ™ or ® should be listed if applicable.

Response: Yes, it is revised and added. (see last paragraph under method section)

20. - Do the physicians chart using scribes (see PMID: 30700408; PMID: 27856140), dictation, manual electronic, or paper charting?

Response: EMR (electronic medical record) system was used for provider charting in this study. We revised and added under the method section.

21. - Was the presence of medical students and other learners recorded and factored in?

Response: Yes, our ED provides medical students rotation all year around. However, medical students are not allowed to document under EMR system. Therefore, residents oversee medical students’ patients. We revised and addressed it under the method section.

22. - Were the same attendings enrolled in both groups depending on whether or not they had a resident, or was there no cross-over?

Response: Yes, the same attendings were enrolled in both groups and this is a cross-over comparison study. We revised and added to the method section.

23. - Just to confirm, were all residents emergency medicine residents, or were some off-service residents (eg. internal medicine, ob-gyn, etc.)?
Response: Yes, study included off-service residents (non-EM residents). These non-EM residents were treated as PGY-1 EM residents. We addressed it under the method section.

24. Results
- Was time of shift and staffing levels recorded? Did these differ between groups? If so, changes in nursing staffing could also have impacted the results.

Response: Yes, time of the shift was recorded, however, due to the different length of shifts (e.g. 8h, 9h, 10h, 12h-shifts), this makes it hard to calculate number of patients per shift. Therefore, we use number of new patients per HOUR for productivity measurement. We understand that nursing staff will also affect productivity and efficiency, unfortunately, we did not record nursing staffing levels nor record the experience levels of nursing staff (RN1 versus RN2 versus more advanced RN levels). We revised and addressed it in the limitation section under the discussion. (see fifth paragraph under discussion section)

25. - What was the distribution of the year of resident training (1, 2, 3, etc.)? It would be important to know if it was not balanced between senior and junior residents.

Response: We have balanced distribution of residents with different levels of training. Each month, we have approximately equal numbers of senior EM residents, PGY-1 EM residents, and non-EM residents. We revised under the method section as the following:

“Attendings are typically scheduled with one senior EM Resident (PGY-2 or PGY-3) and one junior Resident (EM PGY-1 or non-EM). In general, teams are composed of one Attending and two Residents working within a fixed geographic location within the ED. Senior and junior residents are balanced when Attendings working with the residents.”

26. Discussion
- Would benefit from a deeper discussion of why they think they found no difference when others have. Compare and contrast with the results of other published studies on the topic including: (PMID: 24578767; PMID: 24672605; PMID: 25972206; PMID: 24238313; PMID: 18973640)

Response: Thanks for reviewer’s valued comments. We reviewed all mentioned references, some studies have been cited in this manuscript (PMID 24578767, PMID25972206), while others have not. We realize that our study required deeper discussion. Therefore, we revised and added a separate paragraph under the Discussion section to further discuss the differences when attending physicians work with trainees. See the following revision (the fourth paragraph under discussion section).

“Many previous studies investigated the impact of trainees on ED Attending performance concluding diverse findings [7,11,18,19]. When patient ED LOS was measured, some studies showed that ED LOS was not affected significantly by the presence of trainees rotating in the ED [11], whereas other studies showed an increase in ED LOS when Attendings worked with Residents [18,19]. An average of 7 minutes of increased LOS was reported with each additional trainee working with a given Attending [19]. In terms of provider productivity, a previous study also revealed no significant difference in number of patients per hour seen by junior EM, senior EM, or off-service Residents [7]. The reasons that our study findings differ from previous reports include: 1) we balanced our samples of Attendings in both groups. Therefore, the changes in their operational performance can be compared at an individual Attending level. In contrast, other studies mainly assess the operational performance at a group level; 2) we used PDT instead of LOS for provider proficiency measurement which may help reduce the effects of other confounding factors (e.g., ED crowding). The use of propensity score matching could further minimize potential biases; 3) the study ED had 1:2 Attending-to-Resident ratio instead of 1:1 ratio staffing. This might affect attending operational performance more significantly than 1:1 teaching model, which could indirectly result in increased Attending productivity (i.e., more providers are available to see patients during the same time interval) with decreased efficiency (i.e., more trainees requiring more supervision per Attending per unit time), which differs from the previous report [7]. “

6. PLOS authors have the option to publish the peer review history of their article ([what does this mean?](https://journals.plos.org/plosone/s/editorial-and-peer-review-process#loc-peer-review-history)). If published, this will include your full peer review and any attached files.


If you choose “no”, your identity will remain anonymous but your review may still be made public.


**Do you want your identity to be public for this peer review?** For information about this choice, including consent withdrawal, please see our [Privacy Policy](https://www.plos.org/privacy-policy).

Reviewer #1: Yes: Marina Boushra

Reviewer #2: No


[NOTE: If reviewer comments were submitted as an attachment file, they will be attached to this email and accessible via the submission site. Please log into your account, locate the manuscript record, and check for the action link "View Attachments". If this link does not appear, there are no attachment files to be viewed.]


While revising your submission, please upload your figure files to the Preflight Analysis and Conversion Engine (PACE) digital diagnostic tool, <https://pacev2.apexcovantage.com/>. PACE helps ensure that figures meet PLOS requirements. To use PACE, you must first register as a user. Registration is free. Then, login and navigate to the UPLOAD tab, where you will find detailed instructions on how to use the tool. If you encounter any issues or have any questions when using PACE, please email us at [figures@plos.org](mailto:figures@plos.org). Please note that Supporting Information files do not need this step.
