# Supplementary material for: Productivity, efficiency, and overall performance comparisons between attendings working solo versus attendings working with residents staffing models in an emergency department: A Large-Scale Retrospective Observational Study
Source: PLoS One. 2020 Feb 5;15(2):e0228719. doi: 10.1371/journal.pone.0228719 (PMC7001986; doi:10.1371/journal.pone.0228719)
Supplement: Attachment — Submitted filename: Response to Reviewers (R2).docx [file pone.0228719.s008.docx]

**AUTHORS RESPONSE TO EDITORS AND REVIEWERS**

As requested, we have included the original letter and comments with our point by point response in red colored font.

PONE-D-19-26595R1
Productivity, Efficiency, and Overall Performance Comparisons Between Attendings Working Solo Versus Attendings Working with Residents Staffing Models in an Emergency Department: A Large-Scale Retrospective Observational Study
PLOS ONE

Dear Dr. Wang,

Thank you for submitting your manuscript to PLOS ONE. After careful consideration, we feel that it has merit but does not fully meet PLOS ONE’s publication criteria as it currently stands. Therefore, we invite you to submit a revised version of the manuscript that addresses the points raised during the review process.

We would appreciate receiving your revised manuscript by Feb 24 2020 11:59PM. When you are ready to submit your revision, log on to https://www.editorialmanager.com/pone/ and select the 'Submissions Needing Revision' folder to locate your manuscript file.

If you would like to make changes to your financial disclosure, please include your updated statement in your cover letter.

To enhance the reproducibility of your results, we recommend that if applicable you deposit your laboratory protocols in protocols.io, where a protocol can be assigned its own identifier (DOI) such that it can be cited independently in the future. For instructions see: <http://journals.plos.org/plosone/s/submission-guidelines#loc-laboratory-protocols>

Please include the following items when submitting your revised manuscript:

- A rebuttal letter that responds to each point raised by the academic editor and reviewer(s). This letter should be uploaded as separate file and labeled 'Response to Reviewers'.
- A marked-up copy of your manuscript that highlights changes made to the original version. This file should be uploaded as separate file and labeled 'Revised Manuscript with Track Changes'.
- An unmarked version of your revised paper without tracked changes. This file should be uploaded as separate file and labeled 'Manuscript'.

Please note while forming your response, if your article is accepted, you may have the opportunity to make the peer review history publicly available. The record will include editor decision letters (with reviews) and your responses to reviewer comments. If eligible, we will contact you to opt in or out.

We look forward to receiving your revised manuscript.

Kind regards,

Andrew Carl Miller
Academic Editor
PLOS ONE


[Note: HTML markup is below. Please do not edit.]

Reviewers' comments:

Reviewer's Responses to Questions

**Comments to the Author**

1. If the authors have adequately addressed your comments raised in a previous round of review and you feel that this manuscript is now acceptable for publication, you may indicate that here to bypass the “Comments to the Author” section, enter your conflict of interest statement in the “Confidential to Editor” section, and submit your "Accept" recommendation.

Reviewer #1: All comments have been addressed

Reviewer #2: All comments have been addressed

2. Is the manuscript technically sound, and do the data support the conclusions?

The manuscript must describe a technically sound piece of scientific research with data that supports the conclusions. Experiments must have been conducted rigorously, with appropriate controls, replication, and sample sizes. The conclusions must be drawn appropriately based on the data presented.

Reviewer #1: Yes

Reviewer #2: Yes

3. Has the statistical analysis been performed appropriately and rigorously?

Reviewer #1: I Don't Know

Reviewer #2: Yes

4. Have the authors made all data underlying the findings in their manuscript fully available?

The [PLOS Data policy](http://www.plosone.org/static/policies.action#sharing) requires authors to make all data underlying the findings described in their manuscript fully available without restriction, with rare exception (please refer to the Data Availability Statement in the manuscript PDF file). The data should be provided as part of the manuscript or its supporting information, or deposited to a public repository. For example, in addition to summary statistics, the data points behind means, medians and variance measures should be available. If there are restrictions on publicly sharing data—e.g. participant privacy or use of data from a third party—those must be specified.

Reviewer #1: Yes

Reviewer #2: Yes

5. Is the manuscript presented in an intelligible fashion and written in standard English?

PLOS ONE does not copyedit accepted manuscripts, so the language in submitted articles must be clear, correct, and unambiguous. Any typographical or grammatical errors should be corrected at revision, so please note any specific errors here.

Reviewer #1: Yes

Reviewer #2: Yes

6. Review Comments to the Author

Please use the space provided to explain your answers to the questions above. You may also include additional comments for the author, including concerns about dual publication, research ethics, or publication ethics. (Please upload your review as an attachment if it exceeds 20,000 characters)

Reviewer #1: (No Response)

Reviewer #2: It would be great if you were able to run additional analysis to address these 2 other questions:
Impact of EM vs. non-EM residents on attending productivity

Response: Thanks for reviewer’s valued comments. Our attending physicians are staffed to work with one senior EM resident and one junior EM resident or non-EM resident during the same working hours. In this study, we reported attending productivity using number of new patients per hour and this is a mixture of new patients seen by either one senior EM resident + one junior EM resident, or one senior EM resident + one non-EM resident. Therefore, we are unable to do a separate report to see the impact of EM vs. non-EM residents on attending productivity. However, we did another separate analysis to only determine the resident productivity and we found that non-EM resident saw an average of 1.7 patients/hour, similar to junior EM resident (1.8 patients/hour). Based on this evidence, we assume that attending productivity have no significant differences when attending working with 1) one senior EM resident and one junior EM resident versus 2) one senior EM resident and one non-EM resident. We revised our manuscript and added a discussion to our limitation. (Page 16, Line 344-6)

Impact on attending and resident productivity when students are present

Response: Again, thanks for reviewer’s valued comment. In the study ED, medical students see patients together with our senior EM residents, but they are not allowed to do any documentation in the EMR system during study period. Senior EM residents report patients to the attending and do the medical documentations. This could potentially affect senior EM resident productivity to a certain level. Unfortunately, due to the lack of medical students’ data, we were unable to report this impact on attending productivity when students are present. We revised our manuscript and added to our limitation to address this. (Page 16, Line 344-6)

Line 87: students [5-7]., not students. [5-7].

Response: Yes, it is revised. (see Page 4, Line 87)

Line 92: This is a loaded statement. The APPs were likely practicing independently, but the residents have to do more steps before disposition: present to attending, attending sees patient, then disposition. It's not really a fair comparison.

Response: Thanks for reviewers’ valued comments and sorry for the confusion. This is truly what we want to address: residents in training might be less efficient than other providers not in training statuses (such as APPs). We revised this sentence and make it clearer. (see Page 4, Line 90-93).

Line 164: Define how ED crowding was determined (i.e. NEDOCS score). Also state how overcrowding thresholds were selected, including references.

Response: Yes, we defined ED crowding using NEDOCS in Appendix (Appendix-1.2) and also added the thresholds of not-crowding, crowding, and overcrowding separately with references (see Appendix with separate references). Details are as the followings:

Appendix 1.2: Detail Variables Explanation

ED Length of Stay (LOS) is defined as the time in minutes beginning at the point the patient is initially arrived and registered into the EMR at the ED and ending at the point that the patient physically leaves the ED indicating closure of that specific encounter.

Provider-to-Disposition time (PDT) is defined as the time interval documented in the EMR beginning at the point when the patient is initially seen and evaluated by a provider and ending at the point when the disposition decision is made.

The number of new patients per hour is defined as the number of new patients assigned to individual providers within a one-hour block (e.g., 0200 to 0259).

ED crowding is measured using the NEDOCS score (National Emergency Department Over-Crowding Study) upon each patient’s arrival to the ED (see below). The definitions of these variables are consistent with those previously published (Welch SJ, Asplin BR, Stone-Griffith S, Davidson SJ, Augustine J, Schuur J: **Emergency department operational metrics, measures and definitions: results of the Second Performance Measures and Benchmarking Summit.** *Ann Emerg Med* 2011, **58:** 33-40.). NEDOCS score equals or less than 100 (NEDOCS≤100) is considered ED not crowding, NEDOCS score less than 140 but greater than 100 (100<NEDOCS<140) is considered ED crowding, and NEDOCS score equal or greater than 140 (NEDOCS≥140) is considered ED overcrowding.

Detail Explanation of NEDOCS Score Calculation

| Variables | Definition |
| --- | --- |
| NEDOCS = 85.8T + 600B + 5.64W + 0.93L + 13.4C -20 | |
| T | The total number of ED patients collected divided by the number of licensed beds at the time a score is calculated |
| B | The number of admitted patients/number of hospital beds at the time a score is calculated |
| W | Longest wait time in hours for patients in the waiting room at the time a score is calculated |
| L | Longest time in hours since registration among boarding patients at the time a score is calculated |
| C | Number of critical care patients at the time a score is calculated. Typically, this is a site-specific variable which usually refers to patients that require one-to-one nursing care. In the study ED, critical care patients are defined as ICU patients and ICU consulted patients including but not limited to patients on mechanical ventilators, receiving tPA, diagnosed with septic shock, critical trauma patients, and patients requiring conscious sedation at the time a score is calculated, etc. |

Line 175: please provide citation for STROBE

Response: Yes, it is added. (see Page 8, Line 176)

Line 204: “more elderly”: The statement is a little misleading. Although statistically significant, its not really clinically significant. The means were 48 vs 47 years of age.

Response: Yes, we deleted “more elderly” since there was no clinically significant value for such comparisons. We revised as the following: “It was noted that the attending working solo group saw slightly more high-acuity (ESI-1) patients”. (Page 9, Line 205-206)

Line 205: Fewer high acuity patients seen by solo attendings: This is a little misleading. Most places consider high acuity to be ESI 1&2 (not 1-3). The solo patients saw MORE ESI 1 (highest acuity) patients.
All high acuity patients are seen by an attending regardless of resident presence. There is a work-flow nuance here. On shifts with residents, there are likely fewer attendings, and on attending only shifts there is likely more attending coverage. The attending overseeing residents is likely to get more High Acuity patients because multiple residents are picking them up and presenting them. He/She can manage more at 1 time. On the attending only shifts, they are likely to divide up these patients, so any one attending will have fewer than if they were alone with residents. Please discuss this.

Response: Sorry for the confusion. We revised the result and addressed more accurately as the following: “It was noted that the attending working solo group saw slightly more high-acuity (ESI-1) patients”. This could possibly be due to: 1) the daily variety of high acuity patients present at the study ED (e.g. slightly more ESI-1 patients present during non-resident shifts); and 2) the exclusion of the APPs in this study. During the ED non-resident time (16h/week), an extra APP was scheduled to work. However, this study excluded APPs who usually only saw low-acuity patients. This is the reason that propensity score matching comparison was used in the study to minimize these confounders. We also revised and addressed it in our discussion section. (See Page 13, Line 279-283).

Table 1: what characterized ED overcrowding? Was it a NEDOCS score ≥ level 4?
For tables, please use superscript letters a for legends rather than symbols.

Response: Yes, ED overcrowding was addressed in detail in Appendix. NEDOCS score >140 is considered overcrowding. In addition, we revised and used superscript letters for legends (See revised Table 1 and 2).

7. PLOS authors have the option to publish the peer review history of their article ([what does this mean?](https://journals.plos.org/plosone/s/editorial-and-peer-review-process#loc-peer-review-history)). If published, this will include your full peer review and any attached files.


If you choose “no”, your identity will remain anonymous but your review may still be made public.


**Do you want your identity to be public for this peer review?** For information about this choice, including consent withdrawal, please see our [Privacy Policy](https://www.plos.org/privacy-policy).

Reviewer #1: Yes: Marina Boushra

Reviewer #2: No


[NOTE: If reviewer comments were submitted as an attachment file, they will be attached to this email and accessible via the submission site. Please log into your account, locate the manuscript record, and check for the action link "View Attachments". If this link does not appear, there are no attachment files to be viewed.]


While revising your submission, please upload your figure files to the Preflight Analysis and Conversion Engine (PACE) digital diagnostic tool, <https://pacev2.apexcovantage.com/>. PACE helps ensure that figures meet PLOS requirements. To use PACE, you must first register as a user. Registration is free. Then, login and navigate to the UPLOAD tab, where you will find detailed instructions on how to use the tool. If you encounter any issues or have any questions when using PACE, please email us at [figures@plos.org](mailto:figures@plos.org). Please note that Supporting Information files do not need this step.
